# Dispersive Solid Phase Extraction for the Analysis of Veterinary Drugs Applied to Food Samples: A Review

**DOI:** 10.1155/2017/8215271

**Published:** 2017-10-18

**Authors:** Gabriela Islas, Israel S. Ibarra, Prisciliano Hernandez, Jose M. Miranda, Alberto Cepeda

**Affiliations:** ^1^Área Académica de Química, Universidad Autónoma del Estado de Hidalgo, Carretera Pachuca-Tulancingo Km 4.5, 42076 Pachuca, HGO, Mexico; ^2^Área de Energías, Universidad Politécnica de Francisco I. Madero, Domicilio Conocido, 42640 Tepatepec, HGO, Mexico; ^3^Departamento de Química Analítica, Nutrición y Bromatología, Facultad de Veterinaria, Universidade de Santiago de Compostela, Pabellón 4 Planta Baja, Campus Universitario, s/n, 27002 Lugo, Spain

## Abstract

To achieve analytical success, it is necessary to develop thorough clean-up procedures to extract analytes from the matrix. Dispersive solid phase extraction (DSPE) has been used as a pretreatment technique for the analysis of several compounds. This technique is based on the dispersion of a solid sorbent in liquid samples in the extraction isolation and clean-up of different analytes from complex matrices. DSPE has found a wide range of applications in several fields, and it is considered to be a selective, robust, and versatile technique. The applications of dispersive techniques in the analysis of veterinary drugs in different matrices involve magnetic sorbents, molecularly imprinted polymers, carbon-based nanomaterials, and the Quick, Easy, Cheap, Effective, Rugged, and Safe (QuEChERS) method. Techniques based on DSPE permit minimization of additional steps such as precipitation, centrifugation, and filtration, which decreases the manipulation of the sample. In this review, we describe the main procedures used for synthesis, characterization, and application of this pretreatment technique and how it has been applied to food analysis.

## 1. Introduction

The analysis of veterinary drugs has been a recurrent practice in recent years to ensure food quality and minimize the risks that some chemical compounds could present to human health. Their indiscriminate application in therapeutic and prophylactic purposes can lead to residues in animal tissues and in derived foodstuffs [1, 2], inducing problems when consumed such as allergic reactions, hypersensitivity, development bacterial resistance, and in some cases death [3–5].

For these reasons, international organizations, such as the European Commission (EC) and the Food and Drug Administration (FDA), have established maximum residue limits (MRLs) of substances in food samples that are employed for veterinary purposes. The MRL is defined as the acceptable concentration of a substance found in foods of animal origin that are consumed by humans and do not constitute any health risk. In order to prevent the problems caused by excessive use of veterinary drugs, the development of sensitive and robust analytical methodologies has been necessary for the determination of antibiotics residues at *μ*g Kg^−1^ or *μ*g L^−1^ levels in foods of animal origin [6–8].

Several analytical methodologies have been developed for the extraction, isolation, clean-up, and preconcentration of residues of veterinary drugs such as liquid-liquid extraction (LLE) [9], solid phase extraction (SPE) [10], solid phase microextraction (SPME) [11], microwave-assisted extraction (MAE) [12], and pressurized liquid extraction (PLE) [13]. However, the use of these techniques in some cases require additional pretreatments or specific manipulations. The classical technique employed for preconcentration and clean-up in the analysis of veterinary drugs is SPE. This technique has allowed the development of alternative methodologies such as dispersive solid phase extraction (DSPE), which is based on the addition of a sorbent directly into the analytical solution followed by dispersion favoring the contact between the sorbent and the analytes [14, 15]. Once the dispersion process is completed, the sorbent, with the analytes retained on its surface, is separated by a mechanical process, such as centrifugation or filtration. The most attractive property of DSPE is the reduction in sample treatment time that allows more samples to be analyzed in a shorter period of time, in addition to simplicity, adaptability, and easy handling in comparison with the traditional techniques [16].

Since its invention, DSPE has been accepted and applied as a clean-up technique due to its versatility, selectivity, and robustness. It has been used in the extraction, isolation, and clean-up of several compounds present in complex matrices, as in the analysis of veterinary drugs, such as anthelmintics [17–19], benzimidazoles (BZDs) [20], nitroimidazoles [21], sulfonamides (SAs) [22, 23], quinolones (QNs) [24], tetracyclines (TCs) [25], nonsteroidal anti-inflammatory drugs (NSAIDs) [26, 27], and *β*2-agonists [28, 29] present in animal tissues, foodstuffs, lacteous products, and water. Additionally, DSPE has been coupled with several instrumental techniques in the determination and quantification of veterinary drug residues in food samples, such as capillary electrophoresis (CE) coupled with diode-array detection (DAD) [30, 31], ultraviolet detection (UV) [32], and mass spectrometry (MS) [20]; high performance liquid chromatography (HPLC) coupled with ultraviolet detection (UV) [33], fluorescence (FL) [34], and diode-array (DAD) detection [35]; liquid chromatography (LC) coupled with mass spectrometry (MS/MS) [36]; ultra-high performance liquid chromatography (UHPLC) coupled with negative electrospray ionization tandem mass spectrometry (ESI-MS/MS) [37], MS/MS [38], DAD [39], and FL [40]; and ultra-fast liquid chromatography (UFLC) coupled with tandem quadrupole mass spectrometry (MS/MS) [23].

## 2. Background and History

**Figure 1:**
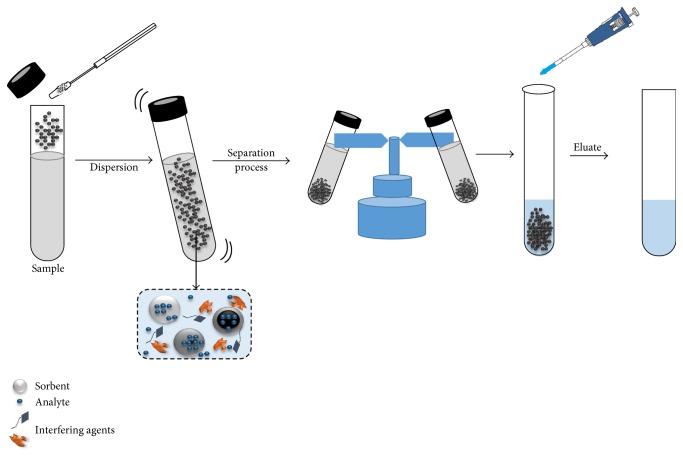
Scheme of dispersion methodology by dispersive solid phase extraction.

Dispersive solid phase extraction (DSPE) has been a widely used technique since its invention around 2000 [41] and has been successfully applied as a method of extraction, isolation, and cleaning in the analytic treatment of a wide variety of veterinary drugs employed in the livestock industry. DSPE simplifies SPE clean-up, allows more samples to be analyzed at one time, is quite rapid, and requires low solvent consumption. DSPE consists of the addition of a solid sorbent, usually silica or polymer based, directly into the sample solution [14–16]. The dispersion process increases the contact area between the sorbent and the analyte. The sorbents employed in DSPE in the determination of antibiotic residues are solids chemically modified by the addition of several chemical compounds that modify their affinities. These modifications ensure the selectivity for the analytes of interest, which allows the maximal retention, minimizing the interferences in the analytical matrix [28]. After the dispersion, the sorbent is isolated by a centrifugation or filtration process. Once the solid phase is isolated, the analytes or interferences adsorbed on the surface of the sorbent could be easily eluted or eliminated with the addition of adequate organic solvents.  Figure 1 shows a scheme of the DSPE procedure [22, 42–47].

DSPE is considered to be a micro- and macroscale method of extraction and cleaning, employed in different analytical methodologies as a procedure for the elimination of potential interferences (clean-up) that could affect the subsequent determination of the analytes [45, 48]. However, one of the critical steps in DSPE is the selection of the sorbent, and it is necessary to consider chemical and physical characteristics that allow maximal interaction between the sorbent and the analytes, ensuring selectivity extraction, removal, or preconcentration of analytes present in analytical matrices [45]. DSPE technique achieves adequate limits of detection (LOD) for the analysis of antibiotics, with the additional advantage of low consumption of solvents in the treatment of the sample. Therefore, it is considered to be a low-cost technique in comparison with classical techniques such as LLE and SPE [22, 44, 47, 49].

The sorbents employed in DSPE are mainly based on silica embedded with several functional groups, such as Supelclean PSA (ethylenediamine-N-propyl) [38, 40, 50, 51], Supelclean-C_18_ (octadecyl, C_18_), -ethylsilane (C_2_), -aminopropyl (NH_2_) [45, 52], that have been previously applied to extraction, clean-up, and even preconcentration process. In this sense, sorbents based on silica are the most used in the extraction process of compounds over a wide range of pH or in the elimination of interferences present in the food matrix such as organic compounds, dyes, lipids, and proteins [45, 50–52]. On the other hand, sorbents as Z-Sep Supelco and Z-Sep+ have been employed to enhance sample clean-up for complex matrices, their Lewis-acid, Lewis-base interactions, allowing the effective removal of fat and color from sample extracts in comparison with the traditional phases for Quick, Easy, Cheap, Effective, Rugged, and Safe (QuEChERS). This kind of sorbents allows obtaining robust analysis and in some cases could replace the conventional sorbents in several methodologies without additional method development [53–55].

**Figure 2:**
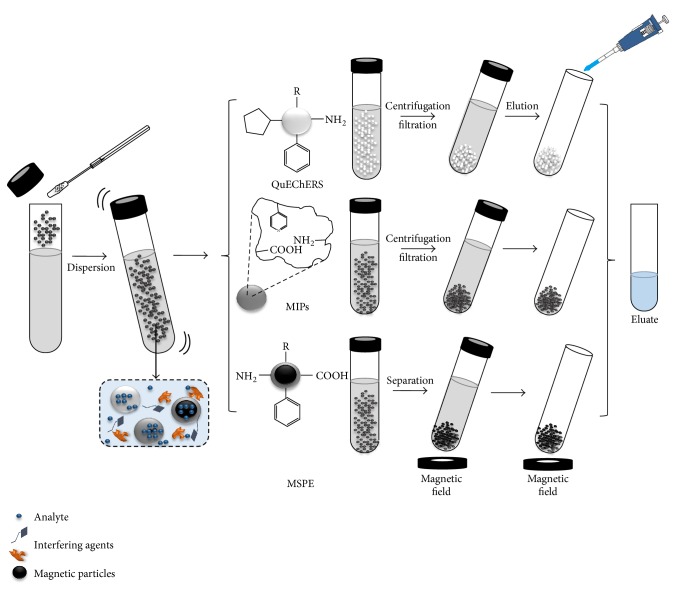
Scheme of dispersion solid phase extraction applied for different techniques.

These sorbents have been applied in the analysis of several drug residues. In recent years, in order to simplify sample pretreatment and minimize organic solvent consumption, several sorbents have been developed and synthesized with higher affinity, selectivity, and retention capacity. These alternatives in extraction, isolation, and clean-up processes are based on the fundamental principle of DSPE, the dispersion of a sorbent in a sample in solution. These analytical methodologies are QuEChERS [50, 53–56] dispersive micro-solid-phase extraction (DMSPE) [57, 58]. The use of sorbents employed in DSPE coupled with magnetic particles has been denominated as magnetic solid phase extraction (MSPE) [30, 31, 59, 60]. Recently, carbonaceous materials have been used as part of the sorbents composition [24, 37, 39, 58], and molecular imprinted polymers (MIPs) have been the techniques most applied on the pretreatment sample in the antibiotic and drug analysis.  Figure 2 shows the basic principles of these techniques, which have been applied successfully for the determination of drug residues [61, 62].

## 3. Solid Sorbents: Types and Characteristics

In recent years, several sorbents have been developed based on the principle of DSPE and applied to several fields, such as biomedicine, biotechnology, materials science, nanotechnology, and polymer synthesis. These sorbents were synthesized by different processes such as sol-gel process, suspension, precipitation, multistep swelling, and bulk polymerization [32, 36, 59, 63]. This new generation of sorbents has been synthesized and applied to MIPs in complex matrices such as agriculture-food samples. The development, design, and synthesis of MIPs are in function of polymerization methods, functional monomer, cross-linking agent, template, porogenic solvent, and the initiator [63–66]. The interactions type according to their structural characteristics are principally electrostatic interactions, hydrogen bonding, Van der Waals forces, hydrophobic and hydrophilic interactions, and dipole-dipole bonds [67–73].

In recent decades, the application of polymers has been increasingly developed to make new alternatives that allow the easy isolation of these particles. Since 1988, paramagnetic materials have been implemented in the process of synthesis of sorbents; these solids are mainly obtained by the partial oxidation of iron in basic media [74]. Generally, the magnetic sorbent is obtained by the encapsulation of inorganic magnetic particles with silica and polymeric and carbonaceous materials [18]. This technique is based on the dispersion of a magnetic adsorbent in solution. The magnetic adsorbents with the analytes adsorbed on the surface can be isolated and eluted with the addition of appropriate solvents. This type of technique has been named magnetic solid phase extraction (MSPE).

MSPE sorbents offer the principal advantage in their easy manipulation in the isolation by the application of an external magnetic field without additional steps, like centrifugation and filtration used in traditional techniques. These characteristics provide advantages in the pretreatment process, making it easy, fast, and low-cost, which minimizes the possible loss of the analyte [34, 75–77]. Carbonaceous materials used in DSPE possess special characteristics in their morphology, such as high surface area, particle size, and sorption capacity. These characteristics and their hydrophobic surfaces have allowed their application in several clean-up processes, extraction, and recently in the preconcentration of some analytes under specific conditions [24]. The carbonaceous materials most commonly used in DSPE are carbon nanotubes (CNTs) [24, 38], activated carbon (AC) [38], graphene (GP) [78], and graphene oxide (GO) [58]. Their sorption capacity depends on the analyte nature (special array, size, and their chemical composition). However, using carbonaceous materials, the retention capacity could be affected by the excess of interferences in the analytical matrix [26, 78].

## 4. Application of Dispersive Solid Phase Extraction: Analysis of Veterinary Drugs in Food Samples

The application of DSPE is based on physical and chemical properties of the sorbents and depends on the technique of synthesis employed. It has been used for the extraction and determination of several veterinary drug residues.  Table 1 shows several molecular recognition sorbents as pretreatment methods in the determination of anthelmintics [18], clenbuterol (CLB) [79], fluoroquinolones (FQs) [80, 81], quinolones (QNs) [82], sulfonamides (SAs) [23, 83], tetracyclines (TCs) [75], and *β*-agonist [34, 73], in both environmental water and food samples [81]. Magnetic solid phase extraction (MSPE) uses a template molecule in the synthesis process, which provides selective mechanisms of recognition based on hydrogen bonding, Van der Waals forces, and dipole-dipole interactions [78–83]. MSPE allows an extraction process that is effective in comparison to the sorbent employed in traditional DSPE, minimizing, in most cases, the number of sample pretreatment steps. MSPE demonstrated a higher affinity for the template molecule allowing obtaining limits of detection in the order of ng mL^−1^ with a % recovery of 63.3 and 124.4 in function of the determination system in the analysis [68, 83].

Different from traditional DSPE, MSPE employs an external magnetic field in the extraction process.  Table 2 shows important aspects in the use of magnetic particles under MSPE methodology, which is usually employed as a procedure for the extraction, isolation, and preconcentration of analytes from large volumes, eliminating additional steps such as centrifugation, precipitation, or filtration of the sample in comparison with the traditional techniques [31, 84].

MSPE was employed for first time in experiments with copper phthalocyanine dye attached to silanized magnetite and magnetic charcoal as sorbents in the separation of safranin O and crystal violet, with an enrichment of up to 460-fold [31, 32, 36]. Their synthesis process involves the obtaining of magnetite which is coated with silica by the reaction of an alkoxy silane in alkaline solution that allows obtaining sorbents with different morphology and particles size in the order of *µ*m until nm [31].

The method has been used in the extraction and isolation of bisphenol A [33], BZD [32], estrogen [35], NSAIDs [29], QNs [30], SAs [36, 85], and TCs [31] in different matrices such as milk or pork. Subsequently at the isolation process, the analytes can be eluted through organic solvents such as acetonitrile, acetone, or methanol, depending on the solubility of each analyte [30, 31, 85]. The % recovery obtained by these methods ranges between 74.0 and 115.6%, with limits of detection in the order of *μ*g L^−1^ and ng g^−1^ in their analysis with techniques such as HPLC and CE using UV, DAD, and MS detectors [30, 31, 85].

Table 3 shows the application of dispersion techniques such as DSPME (dispersive solid phase microextraction) [25], DMSPE (dispersive micro-solid-phase extraction) [57], UAE (ultrasound-assisted extraction) [29], and DSPE (dispersive solid phase extraction) with the principal difference in the use of sample volume in the order of mL and *µ*l during the dispersion step. These types of DSPE employed sorbents coated principally with silica-based C_18_ and primary and secondary amines for the elimination of interferences present in the food matrix. These sorbents allow selective interactions between them and the analyte [12, 52].

However, this process could be affected by interferers present in the analytical matrix, minimizing in some cases the percent retention and subsequent percent recovery. For these reasons, in some cases, additional clean-up steps are necessary to diminish the amount of interferers before analysis of benzimidazole anthelmintic (BAs) [33], bisphenol A [52], nitroimidazoles [21], nitrofurans [86], chloramphenicol [86], QNs [23, 29, 57], TCs [43], SAs [22], and *β*-lactams [45]. In the cited works, limits of detection were obtained in the order of 0.03–123 *μ*g kg^−1^ and 0.7–35.5 ng mL^−1^ in function of the sample, with % recovery of 58% in the analysis of *β*-lactams until 116% for nitroimidazoles, nitrofurans, and chloramphenicol.

Other types of sorbents employed in DSPE are based on the addition of carbonaceous materials in the synthesis process. The use of carbonaceous materials such as multiwalled carbon nanotubes (MWCNT) and graphene oxide (GO) has allowed the development of new class of sorbents. The use of MWCNT commercial with different internal diameters and lengths and the use of grapheme (GP) as precursor in the obtaining of GO by Hummers methods and the preparation of OMWCNT allow strong *π*-*π* interactions with analytes that possess doubles bonds, ensuring an adequate analytical selectivity. The amount of these materials in many cases is limited by the cost and the fact that their synthesis method is not always reproducible. For those reasons, the use of carbonaceous materials is usually employed at low amounts [24, 37–39].

Table 4 shows applications focused on the analysis of amantadine, rimantadine, memantine [38], QNs [24], resorcyclic acid lactones [37], and SAs [22, 39, 58] from food samples such as chicken muscle, ground dried feed, pork, milk, honey, and water samples. The use of MWCNT coated with magnetic particles in the extraction of SAs shows a lower % recovery in comparison with the use of MWCNT that shows in most of the cases % recovery around of 89.2–117.9%; this could be possible in that the amount of magnetite diminishes the active sites [22, 58].

Additionally, the use of O-MWCNT in the synthesis process showed a heterogenic behavior with % recoveries ranging from 62.3% to 116% [24]. However the % recovery employing GO was 95.3–98.3 in the extraction of sulphadiazine. This could be explained by the number of analytes present in the system and the interaction via [24, 58]. The use of carbonaceous materials in clean-up process ensures obtaining limits of detection around of 0.15–129 *μ*g kg^−1^ and 0.34–94.0 *μ*g L^−1^ in their coupled techniques such as UHPLC-MS/MS, CE-DAD, UHPLC-DAD, UHPLC-ESI-MS/MS, and optimized angled mode-mismatched thermal lens spectroscopy (OAMTLS) [22, 87, 88].

Table 5 shows the use of the QuEChERS technique. This technique has been employed successfully in the analysis of several compounds. It is considered to be a simple technique and has been focused on the analysis of multiresidues of several antibiotic groups of veterinary use by UHPLC-MS/MS and CE-MS in ovine muscle, eggs, milk, honey, fish muscle, chicken muscle, and feedstuffs; the QuEChERS method combines a first simple extraction phase and a second solid phase dispersion step, which allows the removal and clean-up of the analytical matrix using PSA sorbent in the clean-up system. It is considered to be a selective method, quick and cheap in comparison with sorbents modified that require specific synthesis processes for each one. This technique takes into consideration the solid characteristics, sample amount/solvent volume ratios, extraction solvent, and pH of extraction; the results obtained show recoveries of % of 56.7–125% with limits of detection between 0.007–220.8 *μ*g kg^−1^ and 3.0–51.0 *μ*g L^−1^. Despite the results obtained, QuECheERS method offers advantages in sample treatment, minimizing solvent consumption, sample manipulation, and loss of the analyte and it is also considered environmentally friendly. It also allows obtaining of appropriate detection parameters in the analysis of residues of antibiotics, according to techniques and international regulations [15, 88–95].

## 5. Conclusions

The DSPE technique is based on the dispersion process and employs the use of solid sorbents. It has been used for the retention of several antibiotic families, used in veterinary medicine, that are employed in subtherapeutic doses as growth promoters for foods of animal origin destined for human consumption. This technique is considered to be an effective strategy for the extraction, isolation, and clean-up and in a few cases in the preconcentration and analysis of residues in complex matrices. The methodology is employed as a basis for the development and application of analytical methodologies such as QuEChERS and MSPE and minimizes additional steps of centrifugation and filtration compared to other classical methods. In general, the use of dispersive solid sorbents allows more contact between the analyte and sorbent phase, improving the retention of the analyte with the additional advantages of lower solvent consumption and lower cost. In the determination of veterinary drug residues, the use of solid sorbents provides good results in the quantification of antibiotics and provides sensitivity and accuracy sufficient for suitable detection limits. These processes take into account the MRLs established by various international standards.

## Figures and Tables

**Table 1:** Molecularly imprinted polymers applied to dispersive techniques in the analysis of veterinary drugs.

Analyte	Matrix	Polymerization or synthesis method	Mode	Technique	Limit of detection	Recovery	Reference
BAs	Water, honey, and milk	Copolymerization ionic liquid monolith/AMII with DB/initiator AIBN/porogen DMF/removing the residue with MeOH	SCSE-AMIIDB	HPLC-DAD	0.020–0.072 *µ*g L^−1^ (water) 0.035–0.10 *µ*g L^−1^ (milk) 0.026–0.076 *µ*g L^−1^ (honey)	70.2–117.6%	[18]

CLB	Biological fluid (bovine)	Coprecipitation (FeCl_2_-FeCl_3_)/monomer MAA, template tert-butylamine, 2-chloroaniline, cross-linker EGDMA, initiator AIBM, with porogen MeOH:acetic acid	MHIM-DSPE	HPLC-UV	0.085 ng mL^−1^	91.4–105.3%	[79]

FQs	Milk	Copolymerization of active vinyl groups present on the nanomagnetic POSS (Fe_3_O_4_@MI-POSS)/monomer MAA, template enrofloxacin, cross-linker octavinyl POSS	MI-MSPE	HPLC-UV	1.76 and 12.42 ng mL^−1^	75.6–108.9%	[67]

FQs	Milk	Fe_3_O_4_-ethylene glycol, ethylene diamine, ammonium acetate	MI-MSPE	HPLC-UV	1.8–3.2 ng g^−1^	94.0–124.4%	[68]

QNs	Chicken tissue	Polymerization with 2-HEMA/template OFL and hydrophilic monomer, cross-linker EGDMA with porogen MeOH	MIP-MSPD	HPLC-DAD	0.008–0.009 *µ*g g^−1^	82.7–102.0%	[82]

SAs	Chicken breast muscle	CS-NR-Mag-MIP/ultrasound-suspension polymerization with GMA, MMA, DVB/Fe_3_O_4_ magnetite/template SAs@TEPA	DMSPE	UFLC-MS/MS	0.0043–0.033 ng g^−1^	85.0–112.2%	[23]

SAs	Duck and chicken tissues	Copolymerization Fe_3_O_4_@SiO_2_/monomer MAA, cross-linker EGDMA, initiator AIBM, template SMZ	MI-MSPE	HPLC-UV	2.0 mg kg^−1^	63.3–76.5% (duck) 68.7–74.7% (chicken)	[83]

TCs	Egg and chicken tissue	MeOH:acetic acid	MMIPS	LC-MS/MS	0.2 ng g^−1^	72.8–96.5%	[75]

*β*-agonists	Pig tissues (muscle, liver)	Bulk polymerization (monomer MAA/cross-linker EGDMA/initiator AIBM/with porogen MeOH and template RAC)	MISDSPE	HPLC-UV	0.2–0.9 *μ*g L^−1^	83.8–115.2%	[73]

*β*-agonists	Pork and pig liver	Coprecipitation (FeCl_3_-FeSO_4_) in ammonia/monomer AM, cross-linker TRIM, template RAC, initiator AIBM, with porogen MeOH	MMIPS	HPLC-FL	0.52–1.04 ng mL^−1^	80.4–90.0%	[34]

TCs: tetracyclines; CLB: clenbuterol; FQs: fluoroquinolones; QNs: quinolones; SAs: sulfonamides; RAC: ractopamine; BAs: benzimidazole anthelmintics; MAA: methacrylic acid; EGDMA: ethylene glycol dimethacrylate; DB: divinylbenzene; AIBM: azo(bis)-isobutyronitrile; DMF: N,N-dimethylformamide; OFL: ofloxacin; 2-HEMA: 2-hydroxyethyl methacrylate; AMII: 1-allyl-3-methylimidazolium bis[(trifluoro-methyl)sulfonyl] imide; MeOH: methanol; AM: acrylamide; TRIM: trimethylolpropane trimethacrylate; CIP: ciprofloxacin; OTC: oxytetracycline; GMA: glycidyl methacrylate; DVB: divinylbenzene; TEPA: tetraethylenepentamine; POSS: polyhedral oligomeric silsesquioxanes; SMZ: sulfamethazine; CS-NR-Mag-MIP: core-shell nanoring amino-functionalized superparamagnetic molecularly imprinted polymer; MMIPS: magnetic molecularly imprinted polymer; MISDSPE: molecularly imprinted polymer-dispersive SPE; MHIM: magnetic hybrid imprinted polymers; DMSPE: dispersive micro-solid-phase extraction; MI-MSPE; molecularly imprinted magnetic solid-phase extraction; SCSE: stir cake sorptive extraction; MSPD: matrix solid-phase dispersion.

**Table 2:** Magnetic sorbents applied to dispersive techniques in the analysis of veterinary drugs.

Analyte	Matrix	Magnetic material coating	Eluent	Mode	Technique	Limit of detection	Recovery	Reference
Bisphenol A	Milk	Magnetic NPs-nylon 6 composite	MeOH	DMSPE	HPLC-UV	3.05 *μ*g L^−1^	86.0–99.0%	[33]

BZD	Swine tissue	Distillation-precipitation polymerization/Fe_3_O_4_/SiO_2_/poly(MAA-co-EGDMA) composite (mixture of MPS-modified, Fe_3_O_4_/SiO_2_, MAA, EGDMA, AIBN)	CAN-TFA	MSPE	CZE-UV	1.05–10.42 ng g^−1^ (muscle) 1.06–12.61 ng g^−1^ (liver)	81.1–105.4%	[32]

Estrogens	Meat (pork)	Magnetic nanoparticles (MNPs)/synthesis of CTAB-coated Fe_3_O_4_@caprylic acid NPs	MeOH	DMSPE	HPLC-DAD	0.02–0.03 ng mL^−1^	93.3–106.7%	[35]

NSAIDs	Milk, human urine, and water	Magnetic ethylenediamine-functionalized graphene oxide nanocomposite	MeOH	UAMDSPME	HPLC-DAD	0.03–0.1 ng mL^−1^	86.4–109.9%	[26]

NSAIDs	Urinary	2-Aminobenzothiazole was polymerized on Fe_3_O_4_, graphene oxide/Fe_3_O_4_, graphene/Fe_3_O_4_	Methanol/acetonitrile	MSPE	HPLC-DAD	0.07–0.3 *µ*g L^−1^	85.5–90.5%	[78]

QNs	Milk	Emulsion polymerization magnetic octyl-phenyl silica (TMOS and PTMS-C_8_ at different molar ratios)	MeOH-NaOH	MSPE	CE-UV	9.0–12.0 *μ*g L^−1^	74.0–98.0%	[30]

SAs	Milk	Distillation-precipitation polymerization/Fe_3_O_4_/SiO_2_/poly(MAA-co-EGDMA) composite (mixture of MPS-modified, Fe_3_O_4_/SiO_2_, MAA, EGDMA, AIBN, ACN)	Acetone-5% ammonia	MSPE	LC-MS/MS	0.5–49.5 ng L^−1^	87.6–115.6%	[36]

SAs	Milk	Emulsion polymerization magnetic phenyl silica (PTMS and TMOS at different molar ratios)	MeOH-NaOH	MSPE	HPLC-DAD	7.0–14.0 *μ*g L^−1^	81.8–114.9%,	[60]

SAs	Milk	Presynthesized Fe_3_O_4_ nanoparticles (NPs) onto HCP/(HCP/Fe_3_O_4_)	ACN	MSPE	HPLC-DAD	2.0–2.5 ng mL^−1^	84.0–105.0%	[85]

TCs	Milk	Emulsion polymerization magnetic phenyl silica (PTMS and TMOS at different molar ratios)	MeOH-acetic acid	MSPE	CE-DAD	2.0–9.0 *μ*g L^−1^	94.2–99.8%	[31]

ACN: acetonitrile; AIBM: azo(bis)-isobutyronitrile; BZD: benzimidazole; CE: capillary electrophoresis; C_8_: octyltrimethoxysilane; CTAB: cetyltrimethylammonium bromide; DAD: diode array detector; DMSPE: dispersive micro-solid phase extraction; EGDMA: ethylene glycol dimethacrylate; HCP: hypercross-linked polystyrene; HPLC: high performance liquid chromatography; MAA; methacrylic acid; MeOH: methanol; MSPE: magnetic solid-phase extraction; NaOH: hydroxide sodium: NSAIDs: nonsteroidal anti-inflammatory drugs; QNs: quinolones; PTMS: phenyltrimethylsilane; SAs: sulfonamides; TCs: tetracyclines; TFA: trifluoroacetic acid; TMOS: tetramethyl orthosilicate; UAMDSPME: ultrasound-assisted magnetic dispersive solid-phase microextraction; UV: ultraviolet visible.

**Table 3:** Applications of dispersive techniques in the analysis of veterinary drugs.

Analyte	Matrix	Functional group	Eluent	Mode	Technique	Limit of detection	Recovery	Reference
BAs	Milk	C_18_	ACN	DSPE	UHPLC-MS/MS	0.14–1.9 *μ*g kg^−1^ 11.0–123 *μ*g kg^−1^	58.0–116.0%	[19]

Bisphenol A	Bovine milk	C_18_	ACN	DSPE	LC-MS/MS	0.15–0.28 *μ*g kg^−1^	95.0–106.0%	[52]

NIIMs	Aquaculture tissue	C_18_ and Z-Sep	ACN	DSPE	UHPLC-MS/MS	0.07–1.0 *μ*g kg^−1^	77.0–88.0%	[21]

NIIMs, NFs, and CAP	Chicken muscle and egg	C_18_	Ethyl acetate	DSPE	UHPLC-MS/MS	0.03–0.16 *μ*g kg^−1^	86.4–116.7%	[86]

QNs and TC	Chicken and pork muscles	Coprecipitation (Fe_3_O_4_) with terbium and europium ion coated magnetic nanocomposite	EDTA (TC) Mg(NO_3_)_2_ pH 9.8 (QN)	DSPE	UHPLC-FL	0.8–4.0 *μ*g kg^−1^	61.5–102.6%	[40]

QNs	Swine muscle	Primary and secondary amine/ACN extract	MeOH-NaH_2_PO_4_ pH 2.5	DMSPE	HPLC-DAD	7.5–26.3 *μ*g kg^−1^	95.5–111.0%	[57]

QNs and *β*-lactam	Raw cow milk	Primary and secondary amine/extraction solvent mixture of acetonitrile/methanol/McIlvaine buffer pH 6	—	UAE/DSPE	UHPLC-MS/MS	0.2–0.6 ng g^−1^ (QNs) 0.1–0.4 ng g^−1^ (*β*-lactam)	96.0–104.5%	[29]

SAs	Chicken muscle	C_18_/ACN extract	ACN	DSPE	LC-FL	1.0–5.0 *μ*g kg^−1^	90.0–95.0%	[22]

SAs	Milk	Butylamide silica	MeOH-basified	DSPE	CE-DAD	0.05 mg L^−1^	73.0–85.0%	[96]

TCs	Water and milk	Primary and secondary amine, or carbonyl groups/ACN extract	—	DSPME	HPLC-DAD	0.7–3.5 ng mL^−1^ (water) 7.9–35.5 ng g^−1^ (milk)	97.1–104.1%	[25]

*β*-Lactams	Bovine kidney tissue	C_18_	—	DSPE	LC-MS/MS	—	58.0%	[45]

ACN: acetonitrile; BAs: benzimidazole anthelmintics; CAP: chloramphenicol; C_18_: octadecyl; DMSPE: dispersive micro-solid-phase extraction; DSPE: dispersive solid-phase extraction; DSPME: dispersive solid-phase microextraction; FL: fluorescence; HPLC: high performance liquid chromatography; LC; liquid chromatography; MeOH: methanol; NIIMs: nitroimidazoles; NFs: nitrofurans; QNs: quinolones; SAs: sulfonamides; TCs: tetracyclines; UAE: ultrasound-assisted extraction; UHPLC: ultra-high performance liquid chromatography.

**Table 4:** Carbon-based nanomaterials applied to dispersive techniques in the analysis of veterinary drugs in different matrices.

Analyte	Matrix	Silica-based functionalized	Eluent	Mode	Technique	Limit of detection	Recovery	Reference
AT, RT, and MT	Chicken muscle	MWCNT	5% formic acid in ACN	r-DSPE	UHPLC-MS/MS	0.15–0.20 *μ*g kg^−1^	96.8–104.6%	[38]

QNs	Water	O-MWCNT prepared by a slight modification	Acetone-MeOH	DSPE	CE-DAD	28.0–94.0 ng L^−1^	62.3–116%	[24]

RAL	Ground dried feed	MWCNT	Ethyl acetate	DSPE	UHPLC-ESI-MS/MS	0.20–0.29 *μ*g kg^−1^	95.3–107.2%	[37]

SAs	Mineral water	MWCNT/magnetic-MWCNT (Magnetic nanoparticles synthesized by means of a solvothermal process, assembled onto CNTs through an “aggregation wrap” mechanism)	MeOH	DSPE	UHPLC-DAD	32.0 ng L^−1^	61.0–110.0% MWCNT 22.0–77.0% Magnetic- MWCNT	[39]

SAs	Pork	MWCNT	ACN/ammonium acetate	DSPE	UHPLC-ESI-MS/MS	112.0–129.0 *μ*g kg^−1^	89.2–117.9%	[22]

SDZ	Milk and honey	Graphene oxide was synthesized using the modified Hummers method and functionalized with iron oxide nanoparticles via coprecipitation	MeOH/acetic acid	DMSPE	OAMTLS	0.34 *μ*g L^−1^	95.3–98.3%	[58]

ACN: acetonitrile; AT: amantadine; CE: capillary electrophoresis; CNTs: carbon nanotubes; DAD: diode-array detection; DMSPE: dispersive micro-solid-phase extraction; DSPE: dispersive solid-phase extraction; EDTA: ethylenediaminetetraacetic acid; ESI-MS/MS: electrospray ionization tandem mass spectrometry; Mg(NO_3_)_2_: magnesium nitrate; MS: mass spectrometry; MT: memantine; MWCNT: multiwalled carbon nanotubes; OAMTLS: optimized angled mode-mismatched thermal lens spectrometer; O-MWCNT: oxidized multiwalled carbon nanotubes; QNs: quinolones; RAL: resorcylic acid lactones; r-DSPE: reversed-dispersive solid phase extraction; RT: rimantadine; SAs: sulfonamides; SDZ: sulfadiazine; TCs: tetracyclines; UHPLC: ultra-high performance liquid chromatography; UV: ultraviolet detection.

**Table 5:** QuEChERS applied to dispersive techniques in the analysis of veterinary drugs in different matrices.

Analyte	Matrix	Extraction procedure	Clean-up	Technique	Limit of detection	Recovery	Reference
BAs	Milk and ovine muscle	10 g of milk simple + 10.0 mL ACN + 5 g MgSO_4_:NaCl	DSPE	HPLC-MS/MS	2.2–2.08 *µ*g Kg^−1^ (milk)	108.0–106.0%	[89]
		The mixture was shaken and centrifuged; the supernatant was cleaned by DMSO solution			771.0–746.0 *µ*g Kg^−1^ (muscle)	109.0–108.0%	

BZDs	Eggs	3 g of sample + ACN + 4 g of MgSO_4_/1 g of NaCl/1.0 mL of ammonium formate solution (10.3 M, pH 7.5) The mixture was centrifuged and diluted with the standard solution CBZ D3	DSPE PSA/MgSO_4_	CE-MS	3.0–51.0 *μ*g L^−1^	74.0–112.0%	[20]

CAP, TAP, and FF	Milk and honey	2 g sample + 15.0 mL of 1% HAc + 4 g Na_2_SO_4_ + 1 g NaCl shaken and centrifuged. Supernatant with 15 g of C_18_, 0.35 g of C_18_ endcapped sorbents and 0.5 g of QuE Z-Sep^+^	QuEChERS Z-Sep^+^/C_18_	LC-MS/MS	0.02−0.045 ng g^−1^	95.8–100.2% (milk)	[53]
		The supernatant was evaporated and reconstituted in 0.5 mL of 10% CAN/water				95.6–99.3% (honey)	

Multiresidue	Chicken tissue	Extraction by SPE, supernatant was added 500 mg (NH_2_ or PSA), shaken, and centrifuged An aliquot of sample was evaporated and dissolved in ACN/water	DSPE/NH_2_/PSA	LC-MS/MS	0.27–444.0 *μ*g g^−1^	37.0–89.0%	[90]

Multiresidue	Honey	A modified QuEChERS without addition of PSA followed by evaporation under vacuum was employed + 4 g of MgSO4 and 1 g NaCl, shaken and centrifuged and reconstituted into 1.0 mL of ammonium formate in MeOH/water	QuEChERS	LC-MS/MS	0.12–0.74 *μ*g kg^−1^	90.9–104.8%	[91]

Multiresidue	Muscle tissue	5 g of sample + 10.0 mL 2% HAc:ACN + 2 g NaCl + 40 g Na_2_SO_4_ The mixture was centrifuged and reconstituted in ACN/formic acid	QuEChERS/C_18_	UHPLC-MS/MS	0.007–66.715 *µ*g kg^−1^	60.0–120.0%	[92]

Multiresidue	Eggs	10 g of sample + 1% of acetic acid/ACN/0.1 M Na_2_EDTA solution, with 4 g of MgSO_4_/1 g sodium acetate The mixture was centrifuged and diluted with MeOH/formic acid, 0.05%	PSA	UHPLC-MS/MS	2.1–220.8 mg kg^−1^	70.4–94.8%	[51]

Multiresidue	Milk	10 g of sample + 1% of acetic acid in ACN/0.1 M Na_2_EDTA solution, with 4 g of MgSO_4_/1 g of sodium acetate The mixture was centrifuged and diluted with MeOH/formic acid, 0.01%	DSPE/PSA	UHPLC-MS/MS	1.0–4.0 mg kg^−1^	70.0–110.0%	[50]

Multiresidue	Chicken muscle	5 g of the sample + water/1% acetic acid/in a solution of ACN:water 0.5 g of sodium citrate dibasic + 1 g sodium citrate dehydrate and 4 g MgSO_4_ The mixture was centrifuged and diluted with formic acid 0.1% in ACN:water	PSA	UHPLC-MS/S	3.0–6.0 *μ*g L^−1^	70.0–120.0%	[93]

Multiresidue	Fish muscle	5 g of sample + water, in ACN:MeOH solution 4 g of MgSO_4_/1 g of sodium acetate The mixture was centrifuged and diluted with mixture 0.1% formic acid in ACN and 0.1% formic acid in water	—	UHPLC-MS/S	7.5 *μ*g kg^−1^	69.0–125.0%	[88]

Multiresidue	Feedstuffs	2 g of ground sample + MeOH:ACN The mixture was centrifuged and the supernatant was diluted with MeOH:0.1% formic acid	DSPE/PSA	LC-MS/MS	0.42–5.74 *μ*g kg^−1^	56.7–103.0%	[94]

Multiresidues	Bovine muscle	2 g sample + 10.0 mL of ACN/water, shaken, centrifuged and 10.0 mL of hexane/can. Reconstituted with 0.1% formic acid Clean-up: hexane; C_18_; Z-Sep^+^; Z-Sep (only) and Z-Sep + C_18_; C_18_ + hexane; Z-Sep^+^ + hexane; Z-Sep + hexane; Z-Sep + C_18_ + hexane	Z-Sep/C_18_	UHPLC-MS/MS	—	70.0–120.0%	[55]

Multiresidues	Animal tissues	DSPE: 2 g sample + 10.0 mL of ACN/water was shaken and centrifuged The DSPE cleanup with 500 mg endcapped C_18_ sorbent + 1.5 mL water in 2 g sample, the final extracts equivalence of 0.174 g mL^−1^ in ≈7 : 3 ACN/water.	DSPE-EMR-L	UHPLC-MS/MS	—	32.0–140.0% (DSPE)	[15]
		EMR-L: 2 g sample + 10.0 mL ACN/5% formic acid was shaken and centrifuged 5.0 mL of 5 mM NH_4_HCO_2_ was added to the EMR-L tube which contains 1 g of the proprietary material and clean-up with 5.0 mL ACN + 2 g MgSO_4_.				4.0–148.0% (EMR-L)	

NSAIDs	Milk	5 g of sample + 10.0 mL of 5% HAc:ACN, + NH_4_HCO_2_ + 4.0 mL of ascorbic acid 0.02 M in HCl 0.24 M + 5 g Na_2_SO_4_. The mixture was centrifuged and cleaned with C_18_-MgSO_4_ and diluted to 500 *μ*L with 0.1% formic acid	QuEChERS	HPLC-MS/MS	0.4–1.5 *μ*g kg^−1^	78.1–97.1%	[27]

QNs	Honey	1 g sample + 30 mM NaH_2_PO_4_ buffer pH 7.0 + 5% formic acid in ACN The mixture was centrifuged and the supernatant was dried under a stream of nitrogen The residue was diluted with H_2_O/ACN/formic acid	—	UHPLC-MS/S	0.2–4.1 *μ*g kg^−1^	70.1–93.7%	[95]

SAs	Chicken muscle and eggs	5 g sample + 5.0 mL water + 10.0 mL ACN: 1% HAc was shaken and 4 g MgSO_4_ + 1 g NaOAc shaken and centrifuged Cleaning up MeCN-based and supernatant + 300 mg of Z-Sep^+^ and once dried was redissolved with 1.0 mL MeOH/water	QuEChERS/PSA/QuE Z-Sep^+^	HPLC-FLD	4.66–28.33 *μ*g kg^−1^	65.9–88.1%	[54]

*β*_2_-agonist	Meat	5 g sample + 4.0 mL of 0.05 M acetate pH 5.2 + 50 *μ*L of *β*-glucuronidase-arylsulphatase The supernatant with DVB-NVP-SO_3_Na, shaken, centrifuged, and dissolved in 0.4 mL of MeOH/water containing 0.1% formic acid	QuEChERS	LC-MS/MS	0.2–0.9 *μ*g kg^−1^	65.0–100.0%	[28]

ACN: acetonitrile; BAs: benzimidazole anthelmintics; BZDs: benzimidazoles; CAP: chloramphenicol; CBZ: carbendazim; CE: capillary electrophoresis; DAD: diode-array detection; DSPE: dispersive solid-phase extraction; DMSO: dimethyl sulfoxide; DVB-NVP: divinylbenzene/N-vinylpyrrolidone copolymer; ESI-MS/MS: electrospray ionization tandem mass spectrometry (ESI-MS/MS); EMR-L: enhanced matrix removal for lipids; HPLC: high performance liquid chromatography; FL: fluorescence; FF: florfenicol; LC: liquid chromatography; MeOH: methanol; MgSO_4_: magnesium sulfate; MS: mass spectrometry; NaCl: sodium chloride; Na_2_EDTA: ethylenediaminetetraacetic acid disodium salt; NSAIDs: nonsteroidal anti-inflammatory drugs; PSA: primary and secondary amine; QNs: quinolones; SAs: sulfonamide; TAP: thiamphenicol; UHPLC: ultra-high performance liquid chromatography; UV: ultraviolet detection.
